# Nrf2 Signaling and the Slowed Aging Phenotype: Evidence from Long-Lived Models

**DOI:** 10.1155/2015/732596

**Published:** 2015-10-25

**Authors:** Danielle R. Bruns, Joshua C. Drake, Laurie M. Biela, Frederick F. Peelor, Benjamin F. Miller, Karyn L. Hamilton

**Affiliations:** Department of Health and Exercise Science, Colorado State University, 220 Moby B Complex, Fort Collins, CO 80523-1582, USA

## Abstract

Studying long-lived animals provides novel insight into shared characteristics of aging and represents a unique model to elucidate approaches to prevent chronic disease. Oxidant stress underlies many chronic diseases and resistance to stress is a potential mechanism governing slowed aging. The transcription factor nuclear factor (erythroid-derived 2)-like 2 is the “master regulator” of cellular antioxidant defenses. Nrf2 is upregulated by some longevity promoting interventions and may play a role in regulating species longevity. However, Nrf2 expression and activity in long-lived models have not been well described. Here, we review evidence for altered Nrf2 signaling in a variety of slowed aging models that accomplish lifespan extension via pharmacological, nutritional, evolutionary, genetic, and presumably epigenetic means.

## 1. Introduction

The incidence of chronic disease increases with age. Understanding the relationships between the processes of aging and age-related diseases is an important initiative of the National Institutes of Health to improve the health of the aging population [1]. Slowing the aging process limits the burden of age-related chronic disease [2]. Identifying characteristics that slow aging may also provide approaches for preventing chronic diseases. Animals with increased lifespan aid in understanding the aging process by allowing the study of physiological and biochemical adaptations associated with slowed aging. Further, studying characteristics shared among long-lived models provides insight into pathways that are key to slowing the aging process and age-associated chronic diseases.

Lifespan can be extended by genetic, dietary, and pharmacological interventions. Additionally, multiple species have independently evolved long lifespan, including humans and naked mole rats, both of which live more than four times longer than predicted by body size [3]. Some of the earliest discoveries of lifespan extension were single-gene mutations associated with the insulin-like growth factor I (IGF-1) and growth hormone (GH) pathways. These mice, including the Snell dwarf [4], are smaller than their heterozygote counterparts and significantly longer-lived, some by 40% or more compared with controls. Long-term caloric restriction is the most consistent dietary manipulation to extend lifespan and recent evidence suggests that short-term transient nutrition restriction prior to weaning, accomplished by litter enlargement, also increases mean and maximal lifespan in mice [5]. Pharmaceutical manipulation of lifespan is in its infancy, with evidence that rapamycin can extend lifespan in mice [6].

It is well established that oxidant stress increases with age across a variety of tissues, including cardiac [7] and skeletal muscle [8], liver [9], and brain [10], and is associated with a wide variety of chronic age-related diseases including cancer, neurodegeneration, sarcopenia, and cardiovascular disease. Although the oxidative stress theory of aging has received criticism [11], it remains true that oxidant stress is associated with the aging process. In response to oxidative stress, cells upregulate antioxidant pathways, including activation of the transcription factor nuclear factor (erythroid-derived 2)-like 2 (Nrf2), the master regulator of antioxidant defenses and the proposed “master regulator” of the aging process [3]. Further, the therapeutic potential of Nrf2 is well supported in neurodegeneration and cancer (reviewed in [12, 13]), highlighting a role for Nrf2 in attenuating age-related chronic disease. Below, we will review what is known about Nrf2 in four models of lifespan extension: caloric restriction, rapamycin feeding, short-term nutrition restriction, and the Snell dwarf mouse. Further, we will discuss what is known about Nrf2 in the exceptionally long-lived naked mole rat and in humans who show enhanced longevity, with the overall goal of describing Nrf2 signaling in longevity interventions and in naturally occurring models of long life.

## 2. Nrf2 Signaling Basics

A member of the basic leucine zipper transcription factor family, Nrf2, controls both basal and inducible expression of over 200 target genes. When cellular stress is low, Nrf2 is sequestered in the cytoplasm by its involvement in an inactive complex with the actin-binding protein Kelch-like ECH-associated protein 1 (Keap1). Under these conditions, Keap1 targets Nrf2 for ubiquitination and degradation by the 26S proteasome system, resulting in basal low-level expression of Nrf2 [14]. However, when activated, Nrf2 translocates to the nucleus and transcriptionally upregulates its cytoprotective transcriptional program through binding to the antioxidant response element (ARE) in the promoter region of its target genes. Activation by reactive oxygen species (ROS) is the best understood mechanism of Nrf2 activation. Oxidant exposure modifies cysteine residues on Keap1 resulting in conformational changes that protect Nrf2 from targeting for ubiquitination and degradation [15], thus resulting in Nrf2 accumulation and activation. In addition to ROS and electrophilic species, Nrf2 can also be activated by phytochemicals [16–18], as well as various pharmaceuticals (reviewed in [19]) via overlapping and distinct mechanisms.

Nrf2 target genes exhibit antioxidant properties and facilitate cellular responses against xenobiotics. Antioxidant enzymes include NAD(P)H dehydrogenase quinone 1 (NQO1), heme oxygenase-1 (HO-1), peroxiredoxin 1 (Prdx1), superoxide dismutase-1 (SOD-1), and many enzymes involved in glutathione synthesis such as glutathione S-transferases (GSTs) and glutamate-cysteine ligase modifier (GCLM), the rate-limiting step in glutathione synthesis (reviewed in [20]). In addition, the cytoprotective properties of Nrf2 activation extend beyond these classic target genes, as other ARE-containing genes exhibit anti-inflammatory activity [21] and autophagic properties [22] and aid in proteasomal removal of oxidative damaged proteins [23]. Nrf2 also regulates its own expression. Two ARE-like motifs in the 5′flanking region of the Nrf2 promoter are responsible for the induction of Nrf2 upon activation [24], ensuring a feed-forward process with Nrf2 activation promoting its own expression and thus facilitating a profound cellular response to stress.

Studies of Nrf2 knockout animals highlight the importance of Nrf2 in cytoprotection. These animals display a diminished ability to activate prosurvival genes [30] and are more susceptible to a wide range of stressors including hyperoxia, lipopolysaccharide, cigarette smoke, and UV irradiation, as well as various chemical insults [31]. Nrf2 knockout animals display diminished basal activity of Nrf2-regulated antioxidant enzymes [30], as well as inducible targets. In a transcriptional analysis of Nrf2 target genes, treatment with the Nrf2 activator 3H-1,2-dithiole-3-thione (D3T) induced 292 genes in wild type animals, compared to only 15 in Nrf2 knockout mice [32]. Together, investigations of Nrf2 knockout animals demonstrate diminished basal and inducible gene programs, as well as increased sensitivity to cell stresses, and support Nrf2 as an important transcriptional regulator of the cytoprotective program.

## 3. Nrf2 and Aging

The role of Nrf2 in responding to cytotoxic stressors is well defined. However, only within the last few years have studies elucidated how Nrf2 function changes with age and how changes in Nrf2 activity contribute to the aging phenotype. Aging is sufficient to diminish cardiac Nrf2-ARE binding activity [7], and aged Nrf2 knockout mice exhibit decreased expression of antioxidant target genes [8]. Disruptions in Nrf2-Keap1 signaling have been reported in skeletal muscle from sedentary older humans [33] and cardiac muscle from aging rats [7]. Impaired liver Nrf2 transcriptional activity in old rats results in glutathione depletion and significant downregulation of Nrf2-regulated glutathione biosynthetic enzymes [34]. Aged mice show similar losses in cellular redox capacity to those observed in Nrf2 knockout mice [34, 35], suggesting that Nrf2 dysregulation with age may be responsible for the loss of cellular redox status. Diminished Nrf2 target gene expression with age is accompanied by increased muscle ROS production, glutathione depletion, and increased oxidant damage to proteins, DNA, and lipids in both humans [33] and rodents [34]. Therefore, given that Nrf2 activity decreases with age alongside increased oxidant stress, interventions that activate Nrf2 may impact the aging process and longevity.

Support for the role of Nrf2 in regulation of lifespan comes from Nrf2 gain of function and loss of function studies. For example, experimental deletion of the antielectrophilic gene glutathione transferase (*gGsta4*) activated Nrf2 and significantly extended lifespan in mice [36]. This mutation increased electrophilic lipid peroxidation products and increased nuclear Nrf2 activity by 43% and 38% in liver and skeletal muscle, respectively. The authors propose that deletion of this glutathione transferase gene resulted in chronic moderate Nrf2 activation and presumably elevated downstream Nrf2 signaling throughout the mouse lifespan. Studies of the Nrf2 homolog SKN-1 in* Caenorhabditis elegans (C. elegans) *and the* Drosophila* homolog CncC further suggest that Nrf2 may be implicated in longevity processes. Upon activation, SKN-1 and CncC upregulate genes involved in the oxidative stress response, including many orthologs to those regulated by mammalian Nrf2 [37, 38]. Similar to mouse Nrf2 knockouts, SKN-1 mutants show diminished resistance to oxidative stress and shortened lifespan. On the other hand, moderate overexpression of a constitutively active SKN-1 increases lifespan, alongside increased resistance to oxidative stress [37]. Similarly, Keap1 loss-of-function mutations extend the lifespan of male* Drosophila* [38]. Various lifespan extending genetic manipulations in* C. elegans *require SKN-1. Dietary restriction activates SKN-1, and expression of the transcription factor in* C. elegans *neuronal cells is required for longevity to be extended by dietary restriction [39]. The long-lived* daf*-2 (nematode homologue of FOXO) mutant increases lifespan in part through resultant activation of SKN-1 [40]. Together, what is known about Nrf2 and aging, alongside preliminary studies of the role of Nrf2 and SKN-1 in cytoprotection, suggests that loss of Nrf2 is important in age-associated declines in oxidant stress resistance, and perhaps in the aging pathology itself.

## 4. Nrf2 in Long-Lived Models

### 4.1. Naked Mole Rat

The naked mole rat is an exceptionally long-lived species, with a lifespan four times longer than similarly sized rodents, thus making the naked mole rat an important model for longevity studies [41]. Naked mole rats do not have typical lifespan curves in which mortality rates increase with age, but rather they experience few of the biological changes typically associated with aging such as decreased metabolic rate, body composition changes, and declines in genomic and proteomic integrity [42]. Naked mole rats are resistant to age-associated diseases such as cancer [43], cardiac diastolic dysfunction [44], and neurodegenerative diseases [45]. Thus, the naked mole rat represents a naturally occurring, unique model of healthy aging.

Surprisingly, early studies of naked mole rats revealed that they have higher levels of oxidative damage, including lipid peroxidation products, protein carbonyls, and oxidative DNA modification in the liver, compared to shorter-lived mouse controls [46]. Subsequent comparisons of young and old naked mole rats revealed a striking difference between age-associated changes in oxidative stress markers [47]. While macromolecular oxidant damage increases with age in mice, as it does in humans, naked mole rats maintain high levels of oxidative damage throughout their lifespan, similar to those observed in old mice [47]. In fact, few genes show differential expression between young and old animals, as assessed by transcriptome analyses in the brain, liver, and kidney from 4- and 20-month-old naked mole rats [26]. Therefore, while naked mole rats from a young age contain higher levels of oxidative damage than shorter-lived control mice, the typical age-associated increase in damage is blunted in this species, suggesting maintenance of oxidant stress defenses over time.

Naked mole rats also have significantly elevated proteasome quality control mechanisms [25]. The high breakdown and clearance of damaged proteins is suspected to be largely due to increased Nrf2 expression, as Nrf2 regulates the transcription of *α* and *β* subunits of the 26S proteasome, as well as the selective autophagy cargo protein p62 [48–50]. In support of the hypothesized role of Nrf2 in naked mole rat longevity, under nonstressed conditions, naked mole rats have greater protein levels of Nrf2 including nuclear Nrf2, elevated Nrf2-ARE binding activity, and greater expression of Nrf2-regulated enzymes in fibroblasts and liver [51, 52]. These data suggest Nrf2 may be responsible for the heightened quality control mechanisms in naked mole rats and may be associated with their exceptional longevity.

### 4.2. Caloric Restriction

Caloric restriction (CR), a decrease in caloric intake without malnutrition, is the most consistent and robust means to increase lifespan across species, from flies to rodents [53], as well as nonhuman primate models [54]. Additionally, CR imparts slowed aging effects and delays the incidence of age-related disease [55]. Although the mechanisms underlying the effects of CR on longevity remain largely unknown, protection against carcinogenesis [56], reduced insulin/insulin-like growth factor (IGF) signaling [57], and prolonged survival [53] are documented. In addition, CR improves cellular adaptation to stress [58], as evidenced by liver mitochondria isolated from CR rats which show delayed opening of the mitochondrial transition pore upon oxidative challenge [58]. In an elegant study using sera collected from humans practicing long-term CR compared to sera collected from age- and sex-matched individuals following a typical western diet, treatment of cultured human primary fibroblasts with CR sera significantly upregulated gene expression of stress-response genes and enhanced tolerance to oxidants [59]. CR decreases ROS production, enhances the plasma membrane redox system, improves insulin signaling, and attenuates inflammation [27], all of which have been associated with improved age-related disease outcomes.

**Figure 1:**
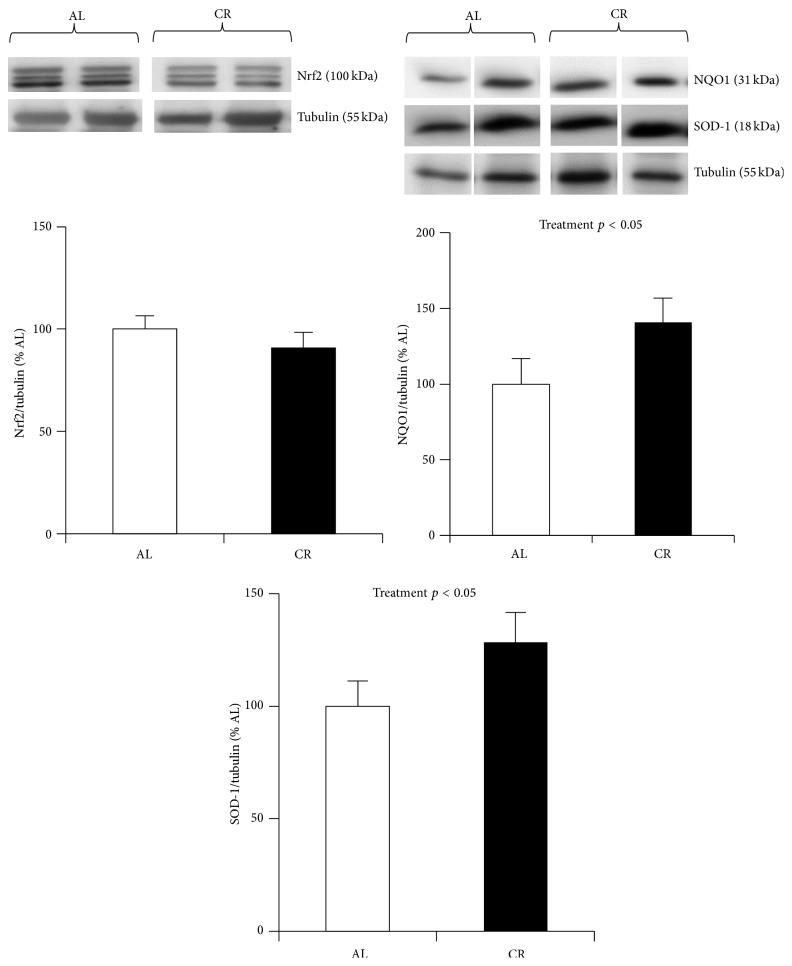
Nrf2 regulated protein expression in cardiac muscle from lifelong calorically restricted mice. Nrf2 was not different compared to ad libitum (AL) mice, while NQO1 and SOD-1 were significantly higher in cardiac muscle from CR mice compared to AL. Nrf2, NQO1, and SOD-1 were analyzed by western blotting and normalized to tubulin, shown below the proteins from each blot. Data are expressed as a ratio of target protein to tubulin (mean ± SEM). *n* = 6 males in each condition. Lifelong B6D2F1 CR mice were maintained at the NIA colony at 40% food restriction compared with AL. Male mice were purchased at 6 months of age and divided into AL and CR groups. CR animals were maintained on NIH-31/NIA Fortified Diet, whereas AL animals were maintained on NIH-31 diet.

Many of the positive outcomes of CR have been associated with Nrf2 activation. For example, a variety of carcinogens activate Nrf2 and the ARE, protecting against carcinogenesis [60]. Changes in insulin levels, such as those elicited by fasting, elicit a small acute oxidant stress and subsequent activation of Nrf2 and its targets [61]. CR prevents the age-induced loss of cellular antioxidant capacity, in part due to increased levels of Nrf2 targets NQO1 and GSTs in brain and liver [62, 63]. In cerebral vascular endothelial cells, CR prevents the age-related decline in Nrf2 activity. Further, CR upregulates Nrf2 expression in old mice to a level that surpasses that of young ad libitum (AL) fed animals [64], highlighting the importance of Nrf2 with aging and its potential activation by longevity promoting interventions. Several proposed CR mimetics, such as resveratrol, quercetin, and curcumin [65], act to increase lifespan and slow aging at least in part through activation of Nrf2 [28, 66–68]. We assessed Nrf2 signaling in cardiac muscle from 7-month-old animals that underwent lifelong 40% reduction in caloric intake compared to mice fed AL. We found Nrf2 protein expression to be unchanged by CR. However, NQO1 and SOD-1 were significantly upregulated (Figure 1) in CR, suggesting that a lifelong reduction in caloric intake may activate Nrf2 and be implicated in longevity in these animals.

Despite the positive associations between CR and Nrf2, the seminal study of Nrf2 and CR-induced lifespan extension is conflicting [69]. At 20 weeks of age, male and female Nrf2 knockout and wild type mice initiated a 40% reduction in caloric intake. As anticipated, the wild type mice were significantly protected against carcinogenesis on the CR diet compared to Nrf2 KO animals. However, survival curves showed that the CR and AL Nrf2 knockout mice overlapped until week 80, when they began to deviate for the remainder of the lifespan study, with CR Nrf2 knockout mice significantly outliving AL Nrf2 knockout mice. Therefore, while CR protected against carcinogenesis in a manner that was dependent on Nrf2, genetic ablation of Nrf2 did not attenuate lifespan extension by CR. This study suggests that lifespan extension in mice by CR is not entirely dependent on upregulation of the Nrf2 pathway, despite its promising role as a chemoprevention target. However, this conclusion is not without criticism, and a role of the antioxidant response in CR-mediated longevity cannot be completely ruled out [29]. Future investigations into whether or not other age-related diseases are slowed or reversed by Nrf2 activation and whether the attenuation of these diseases by CR requires Nrf2 are warranted.

### 4.3. Rapamycin

**Figure 2:**
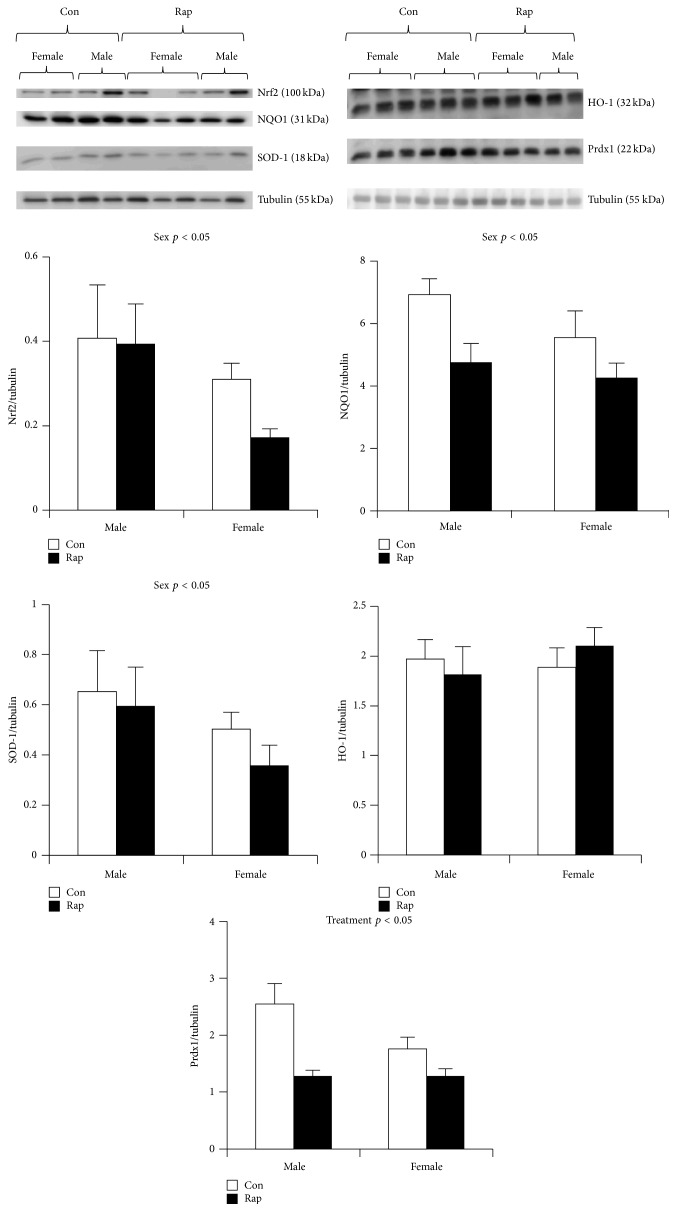
Nrf2 regulated protein expression in skeletal muscle from mice chronically fed with rapamycin. Nrf2, NQO1, and SOD-1 were significantly greater in skeletal muscle from male rapamycin (Rap.) treated mice. Chronic rapamycin feeding suppressed Prdx1 expression compared to controls. Nrf2, NQO1, SOD-1, and Prdx1 were analyzed by western blotting and normalized to tubulin, shown below the proteins from each blot. Data are expressed as a ratio of target protein to tubulin (mean ± SEM). *n* = 6 males and *n* = 6 females in each condition. UM-HET3 mice were generated by the offspring of crosses between (BALB/cByJ x C57BL/6J) F1 females and (C3H/HeJ x DBA/2J) F1 males. Mice were fed with chow mixed with encapsulated rapamycin at 14 mg/kg food (equivalent to 2.24 mg of rapamycin/kg body weight/day) or normal chow for 12 weeks in accordance with the original study describing lifespan extension [6].

Rapamycin, a well-defined inhibitor of the mechanistic target of rapamycin (mTOR), interacts with the Nrf2 signaling pathway, and Nrf2 may be implicated in rapamycin-mediated longevity. Exposure of adult* C. elegans *to rapamycin activates SKN-1 and downstream gene targets and significantly increases oxidative stress resistance in SKN-1 dependent manner [70]. Further, rapamycin treatment increased nematode lifespan through SKN-1, as evidenced by an abolished effect of rapamycin-mediated lifespan extension when SKN-1 was silenced by RNA interference [70]. Treatment with rapamycin also extends lifespan [71, 72] and slows the aging phenotype in mice, including decreased liver degeneration, attenuated cataract severity, and blunting of the age-associated decline in physical activity [73]. In vitro, chronic treatment with rapamycin significantly upregulates Nrf2 protein and transcript expression Nrf2 in fibroblasts, protects cultures from exogenous ROS exposure, and increases replicative lifespan of fibroblasts in culture [74]. We recently assessed skeletal muscle Nrf2 expression, as well as the expression of Nrf2 target proteins NQO1, HO-1, Prdx1, and SOD-1 in mice treated with rapamycin for 12 weeks. We were surprised to find no differences in Nrf2 and Nrf2 target protein expression between the long-lived rapamycin fed mice and controls. Prdx1 expression in skeletal muscle was significantly lower than control, but no other differences were observed in Nrf2 target protein expression between long-lived and control animals (Figure 2). Interestingly, we further assessed sex differences and found NQO1, Nrf2, and SOD-1 to be significantly greater in skeletal muscle from male mice compared to females (Figure 2). Given the disparate lifespan between males and females, and dissimilar effects of longevity interventions on male versus female mice, it is important to understand sex-specific signaling of the Nrf2 cytoprotective pathway, as elucidation of Nrf2 signaling in long-lived males and females may provide insight into the mechanisms behind sexual dimorphic longevity.

### 4.4. Snell Dwarf Mice

Snell dwarf mice are homozygous for a single-gene mutation at the* Pit1* locus. This mutation results in an underdeveloped anterior pituitary and decreases in growth hormone (GH) and insulin-like growth factor-1 (IGF-1) signaling. These mice display a 40% increase in mean and maximal longevity in both male and female mice compared to mice on similar backgrounds [4] and show delay in many age-related pathologies including attenuation of age-dependent collagen cross-linking and age-sensitive indices of immune system status [4]. This single gene mutation thus controls both maximal lifespan and the timing of senescence and age-related pathology, supporting the role of the IGF-1 and GH pathways in regulating mammalian longevity.

**Figure 3:**
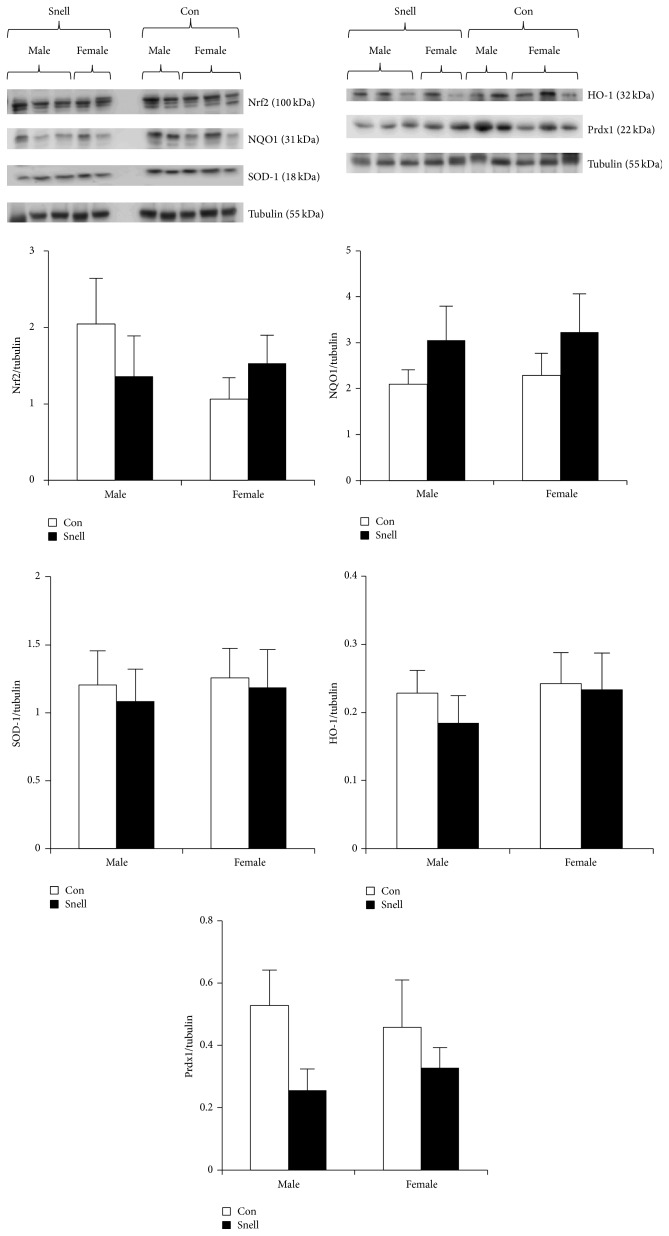
Nrf2 regulated protein expression in skeletal muscle from Snell dwarf mice. The* Pit1 *mutation does not influence Nrf2 or Nrf2 target protein expression in skeletal muscle. Nrf2, NQO1, HO-1, SOD-1, and Prdx1 were analyzed by western blotting and normalized to tubulin, shown below the proteins from each blot. Densitometric analyses were conducted on the multiple band of Nrf2. Data are expressed as a ratio of target protein to tubulin (mean ± SEM). *n* = 10 males and 10 females of each genotype. Snell dwarf (*dw/dw*) and heterozygote (*dw/+*) control mice were bred as the progeny of (DW/J x C3H/HeJ) F1* dw/+* females and (DW/J x C3H/HeJ) F1* dw/dw* males [4].

Snell mice display heightened Nrf2 signaling, as evidenced by increased skin-derived fibroblast expression of total cell Nrf2 protein compared to controls, in addition to upregulated expression of Nrf2 targets HO-1, thioredoxin, and GCLM [75]. In addition, Snell-derived fibroblasts show enhanced resistance to various forms of cytotoxic stress, like paraquat, peroxide, cadmium, and others that kill cells in part via ROS [76]. We recently assessed Nrf2 and targets in skeletal and cardiac muscle from 7-month-old Snell mice and found no differences in Nrf2 and target protein expression compared to control mice (Figure 3) in either males or females. Part of the discordance in Nrf2 signaling in primary fibroblasts compared to skeletal and cardiac muscle may be due to tissue-specific differences. Previous work suggests that Nrf2 activators induce the Nrf2 transcriptional program in some cell types, but not others [19, 77], demonstrating that cell and tissue types respond quite differently to Nrf2 activators. Varying metabolic demands between tissues may be responsible for tissue-specific Nrf2 activity [77], or inherent properties of the tissue, such as expression of Nrf2 activators/inhibitors. An assessment of tissue-specific differences in cellular stress responses is lacking and may play an important role in determining the response of Nrf2 to different longevity interventions.

### 4.5. Crowded Litter

Recent evidence shows that very-short-term nutrient restriction limited only to the preweaning phase by litter crowding extends lifespan by 16% in female mice and 7% in males [5]. In this model termed crowded litter (CL), increasing litter size from eight pups per mother to 12 imposes a transient energy restriction thought to represent an epigenetic means of improving lifespan [78], as the energy stress imposed only in the first three weeks of life extends lifespan and healthspan [79]. At weaning and through adult life, CL mice are leaner and consume more oxygen relative to body mass compared to control mice and have improved lifelong alterations in metabolic status [79]. CL mice have less body fat, lower leptin levels, and higher glucose tolerance and are more insulin sensitive than control mice [79]. Despite few studies interrogating mechanisms by which CL intervention imparts improved healthspan and lifespan, it seems clear that long-lasting endocrine and metabolic effects result from the early-life nutrient restriction.

**Figure 4:**
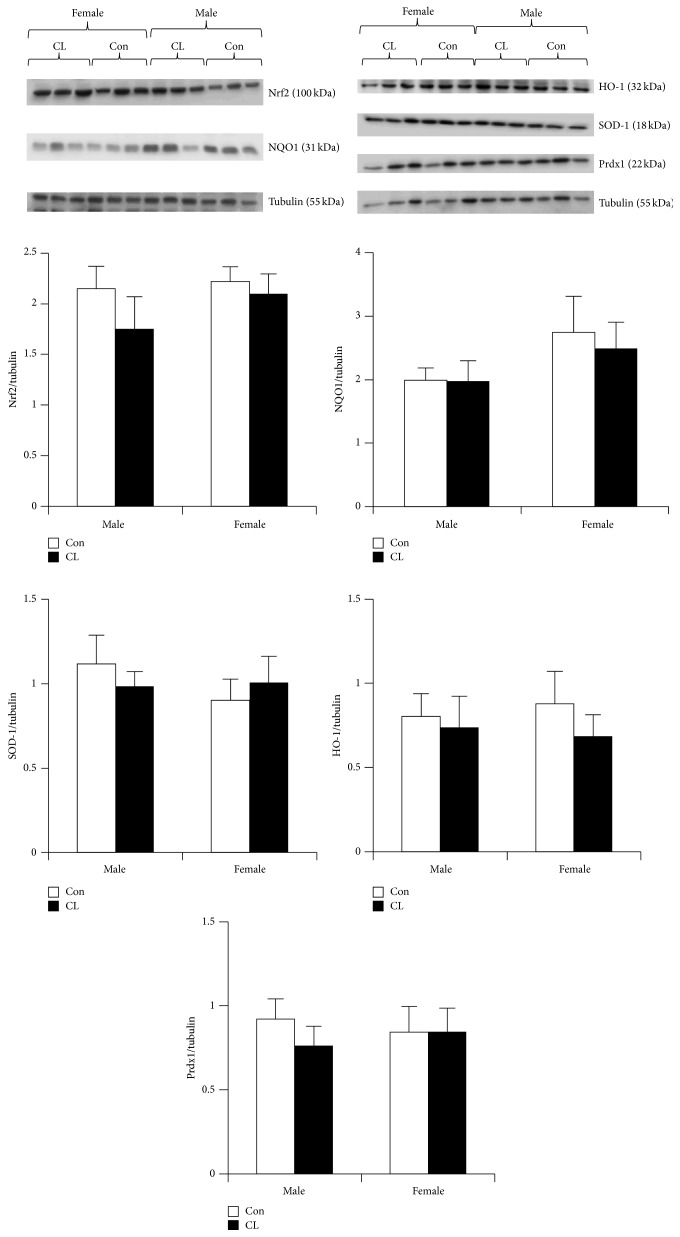
Nrf2 regulated protein expression in skeletal muscle from crowded litter mice. No significant sex or model differences were observed in skeletal muscle from crowded litter (CL) animals. Nrf2, NQO1, SOD-1, and Prdx1 were analyzed by western blotting and normalized to tubulin, shown below the proteins from each blot. Data are expressed as a ratio of target protein to tubulin (mean ± SEM). *n* = 8 males and *n* = 8 females in each condition. CL mice, 3-4 months old, were generated following the previously described procedure [5]. UM-HET3 litters were culled to eight pups (control) or supplemented by transfer of newborn mice to produce litters of 12 mice (CL). Litters were weaned at three weeks of age, after which both groups were fed ad libitum.

As studies of CL mice are in their infancy, minimal data thus far directly link Nrf2 and CL. However, investigations of xenobiotic metabolism and resistance to cytotoxic insult suggest that CL mice maintain elevated levels of xenobiotic phase I metabolizing enzymes in the liver, alongside heightened resistance against* tert*-butylhydroquinone [78], an oxidant known to activate Nrf2 [80]. Based on these data showing enhanced stress resistance in CL, we assessed Nrf2 and targets in skeletal muscle from 3-4-month-old CL mice. In this young cohort, we failed to find differences in Nrf2 targets between CL and control (Figure 4) in either male or female mice. As metabolic and hormonal status change throughout development in the CL mice, corresponding alterations in Nrf2 activation may occur. Given the early stages of this longevity intervention, few investigations have been conducted into developmental changes in CL mice and how metabolism and Nrf2 may interface to regulate longevity in this model. Future investigations are warranted to more completely assess whether Nrf2 mediates lifespan extension and what role Nrf2 may play throughout the lifespan of CL mice.

### 4.6. Humans

Humans are amongst the longest-lived mammals, with a maximum species lifespan potential (MLSP) of over 100 years and a predicted lifespan four times longer than estimated by body mass [42]. Exceptionally long-lived humans, centenarians, may have constitutively upregulated Nrf2, allowing them to better respond to cell stresses and minimize cell damage with age [81]. Investigations of centenarians who seem to reside in areas with high nutrient density and low caloric density diets support this hypothesis, as these diets may be characterized as a prolonged mild form of CR. Physical activity is one of the most effective interventions to prevent chronic disease and delay the detrimental cellular changes with age (reviewed in [82]). Acute exercise stress activates Nrf2 [83] and is associated with enhanced antioxidant capacity. Therefore, it is plausible that exercise, a longevity-promoting intervention in humans, may promote healthspan in part through activation of Nrf2. Nrf2 activators are currently undergoing phase II clinical trials for treatment of various human chronic diseases. Bardoxolone methyl therapy, a therapeutic pulmonary hypertension intervention [84], and dimethyl fumarate (BG-12), a currently approved therapy for relapsing-remitting multiple sclerosis, promote cytoprotective properties, in part through activation of Nrf2 [85]. A sulforaphane-based pharmaceutical is slated to begin trials for prostate cancer [86] following preliminary studies showing diminished Nrf2 signaling in prostate cancer that is restored by sulforaphane treatment [87]. Thus it appears that some of the human diseases associated with aging may be treated with Nrf2 activators. However, whether Nrf2 expression is higher in long-lived centenarians remains unknown. Further, whether Nrf2 activation can increase human lifespan or delay human aging is still unexplored.

### 4.7. Nrf2 Activity and Maximal Lifespan Potential

The simultaneous study of species with varying MLSP facilitates the identification of associations between species longevity and specific mediators of lifespan, such as Nrf2. A comparison of eight rodent species with widely divergent longevities ranging from 4 to 31 years yielded a positive association between Nrf2-ARE binding activity and MLSP [52]. Quantitatively, for a 10-year increase in lifespan, Nrf2 activity increased 1.4-fold. Surprisingly, the authors of this study found that Nrf2 protein levels did not correlate with MLSP. Rather, mechanisms that control Nrf2 activity, such as Keap1 expression, significantly correlated with MLSP. Therefore, the authors posited that long-lived species are poised to effectively respond to cellular stresses associated with age and chronic disease due to the natural variation in Nrf2 activity. Together, these data support the notion that Nrf2 may be the “guardian of healthspan and the gatekeeper of species longevity” [3] and suggest that future investigations should elucidate whether activation of Nrf2 facilitates increases in healthspan and lifespan.

## 5. Mechanisms of Nrf2 Activation: Basal versus Inducible Activity

Our results of Nrf2 in long-lived mice presented here were obtained from young (3-4 and 7 months of age) mice that were not exposed to acute or chronic stresses. Young, unstressed animals generally do not display elevated Nrf2 signaling, as supported by investigations of young Nrf2 knockout mice, which do not have evidence of redox imbalance as assessed by either expression of Nrf2 target genes, overall cellular antioxidant status, or overt macromolecule oxidant damage [83]. However, when these Nrf2 knockout mice undergo an acute stress or when mice age, the ability to respond to and recover from the stress is attenuated. Thus, we suggest that the young mice in the long-lived cohorts reviewed here did not display enhanced Nrf2 signaling because there was no stressful stimulus from which to necessitate enhanced Nrf2 activation. Further, we assessed Nrf2 protein expression, as well as downstream Nrf2 target expression. Previous correlations between Nrf2 protein expression and MSLP, however, show no association between these two measurements [52]. Instead, Nrf2 activity, as assessed by Nrf2-ARE binding activity, along with proteins that influence Nrf2 activity, such as Keap1 expression [38], predicted MLSP. Therefore, we suggest that future investigations of Nrf2-mediated longevity should assess Nrf2 activity and the key proteins that regulate Nrf2 activation and nuclear localization. Further, we propose that these investigations be conducted in aged animals or younger animals administered a stressful stimulus. It is under these conditions that differences in Nrf2 signaling between long-lived and control animals should be most apparent.

## 6. Conclusions and Future Directions

Chronic disease incidence increases with age. Slowing the aging process limits the burden of chronic disease [2]. The transcription factor Nrf2, a proposed “master regulator of the aging process,” regulates a wide battery of cytoprotective responses and helps attenuate age-related disease, and its activity is positively associated with species lifespan potential. Previous work of Nrf2 in long-lived models shows promise for slowed aging interventions (summarized in Table 1), with naked mole rats, an exceptionally long-lived species, showing enhanced Nrf2 signaling compared to shorter-lived mouse species. Caloric restriction and many of its pharmaceutical mimetics activate Nrf2 and protect against age-associated carcinogenesis, despite contradicting evidence showing no effect of Nrf2 expression in CR-induced longevity. Studies of rapamycin treated, Snell dwarf, and CL mice suggest that primary cells from these animals are more resistant to cytotoxic stress, which may be linked to elevated Nrf2 signaling. Future investigations should identify whether aged animals from long-lived cohorts have enhanced Nrf2 signaling that may explain their stress resistance. We hypothesize that pharmaceutical, genetic, epigenetic, and dietary manipulations that extend lifespan will enhance Nrf2 activation at advanced ages and under stressful cellular conditions, contributing to stress resistance and extended healthspan. Further, future investigations of Nrf2 signaling in humans, and the ability of Nrf2 activation to prevent chronic disease associated with aging, will lend further insight into the role of Nrf2 activation as a possible longevity-promoting intervention.

## Figures and Tables

**Table 1:** Summary of Nrf2 expression, Nrf2 target expression, and Nrf2 activity in long-lived models.

Model	Tissue/cell type	Nrf2 expression	Nrf2 target expression (NQOl, HO-1, SOD-1, etc.)	Nrf2 activity (Nrf2-ARE binding)	Reference
Naked mole rat	Liver	↑	↑	↑	[25]
	Fibroblast	↑	↑	↑	[26]

Caloric restriction	Heart	*↔*	↑	N.R.	This manuscript
	CMVEC	↑	N.R.	↑	[27]

Rapamycin	SkM	*↔*	*↔*	N.R.	This manuscript
	*C. elegans *	↑	↑	↑	[28]

Snell	SkM	*↔*	*↔*	N.R.	This manuscript
	Heart	*↔*	*↔*	N.R.	This manuscript
	Fibroblast	↑	↑	N.R.	[29]

Crowded litter	SkM	*↔*	*↔*	N.R.	This manuscript

Humans	—	—	—	—	

Arrows indicate difference in the long-lived model compared to corresponding control, with ↑ indicating “greater in the long-lived model compared to control,” *↔* indicating “no difference between long-lived model and control,” and N.R. indicating “data were not reported in the investigation.” To date, no experimental data for Nrf2 expression or activity have been reported in aged humans. CMVEC: cerebromicrovascular endothelial cells; SkM: skeletal muscle (mixed skeletal muscle: gastrocnemius, soleus, and plantaris).

## Abbreviations

Nrf2: NFE2L2, nuclear factor (erythroid-derived 2)-like 2
SOD-1: Superoxide dismutase-1
NQO1: NAD(P)H hydroquinone oxidoreductase-1
HO-1: Heme oxygenase-1
Prdx1: Peroxiredoxin 1
CL: Crowded litter
CR: Caloric restriction
Rap: Rapamycin
MLSP: Maximum species lifespan potential.
